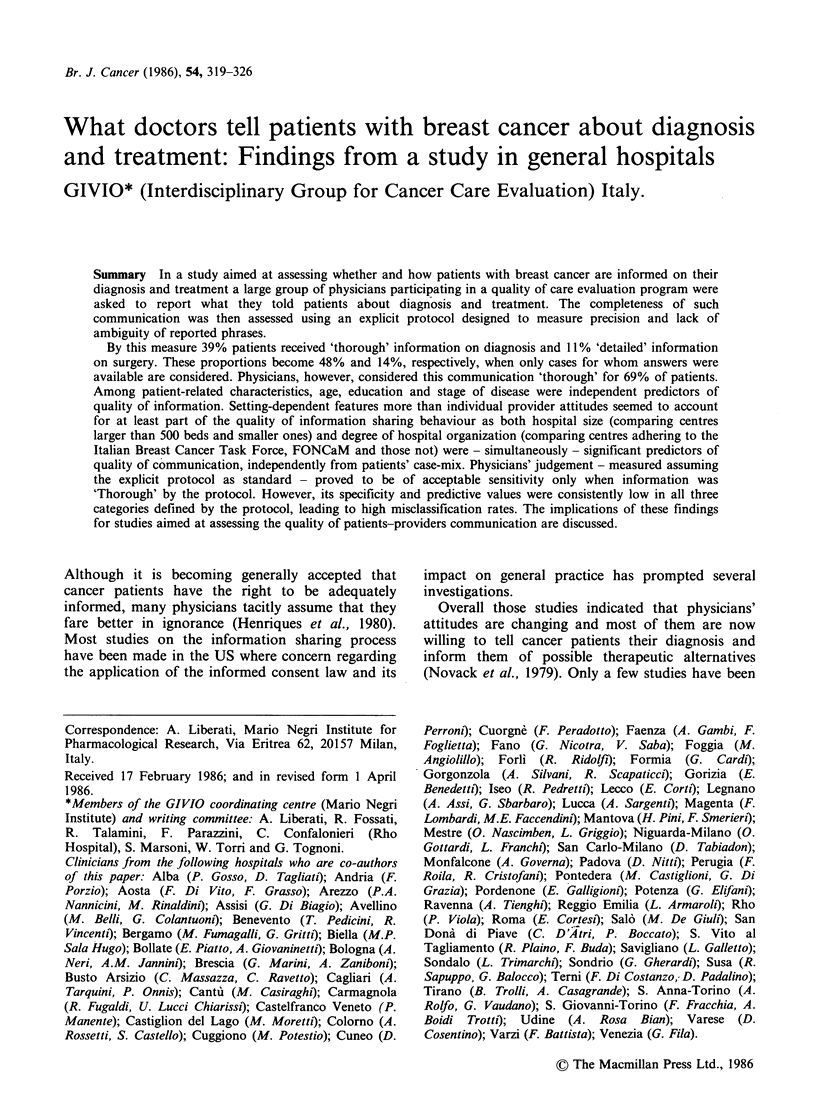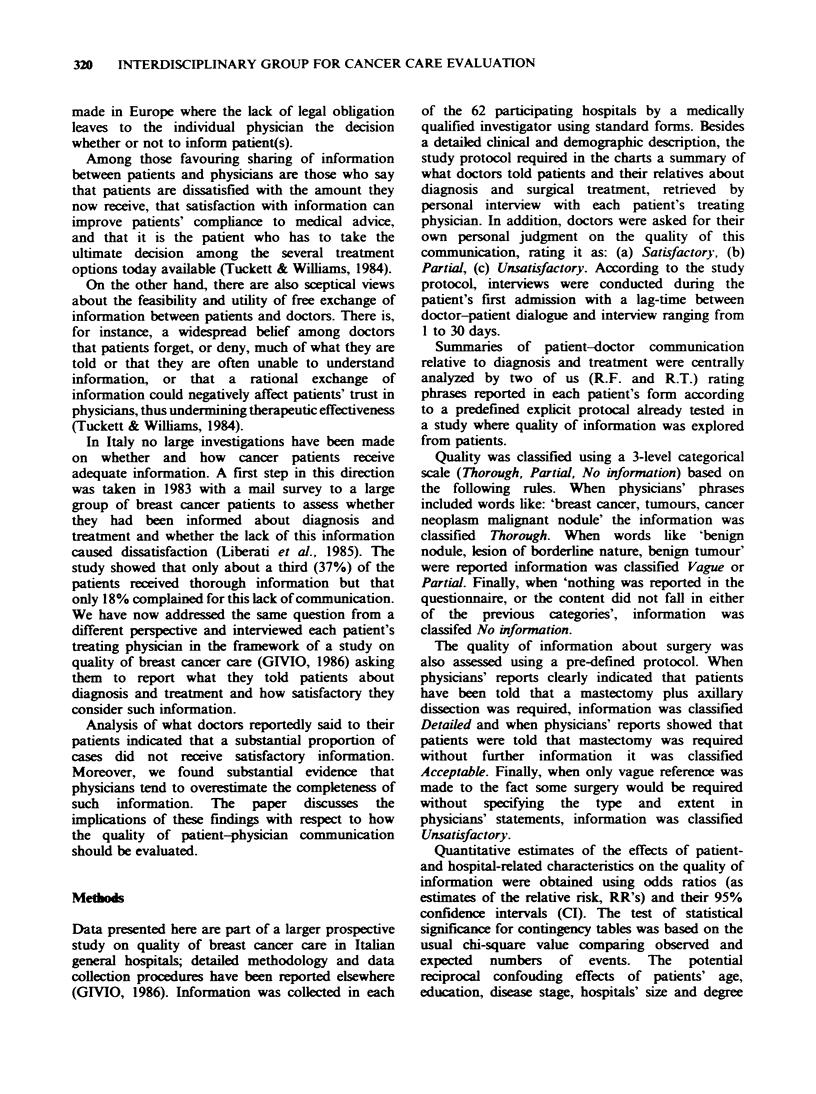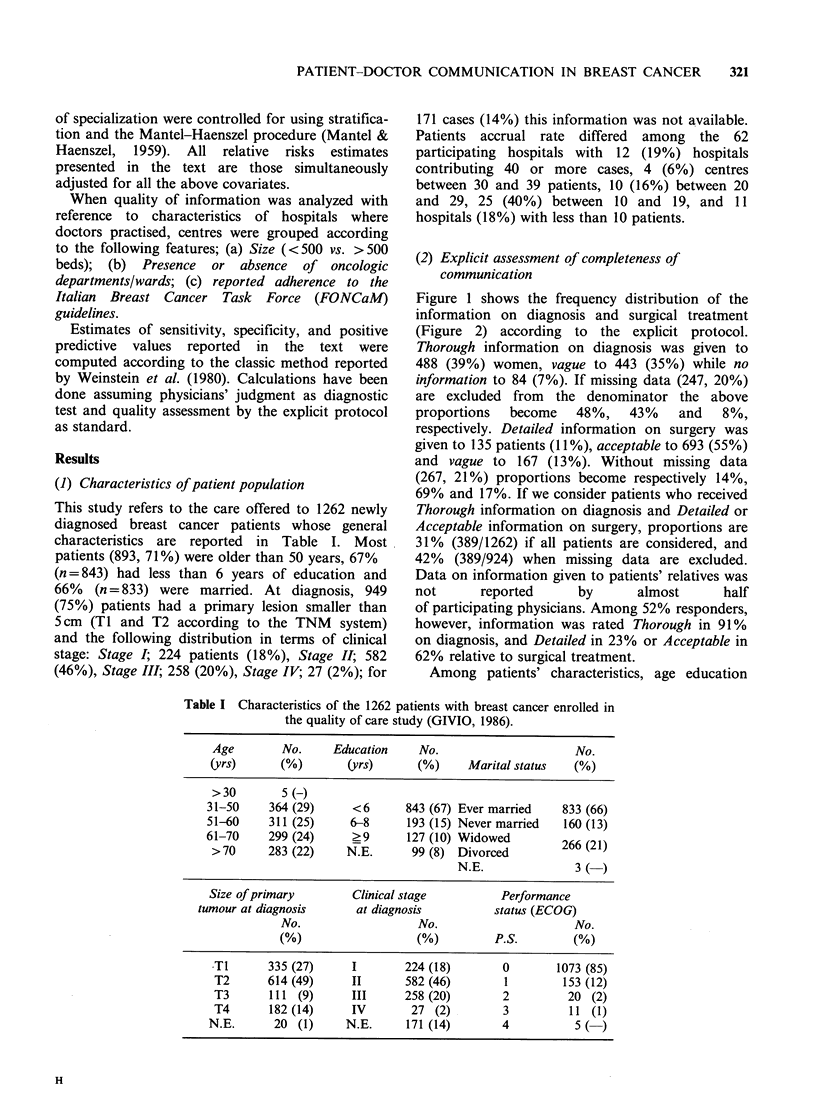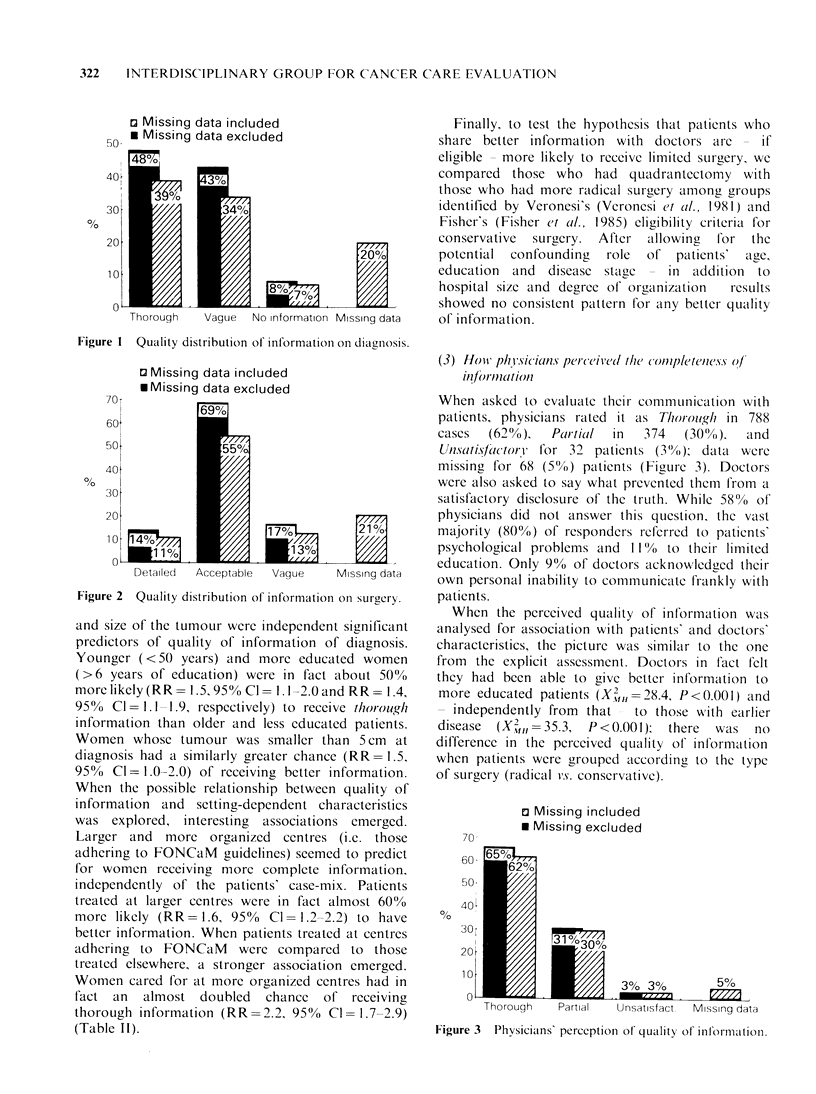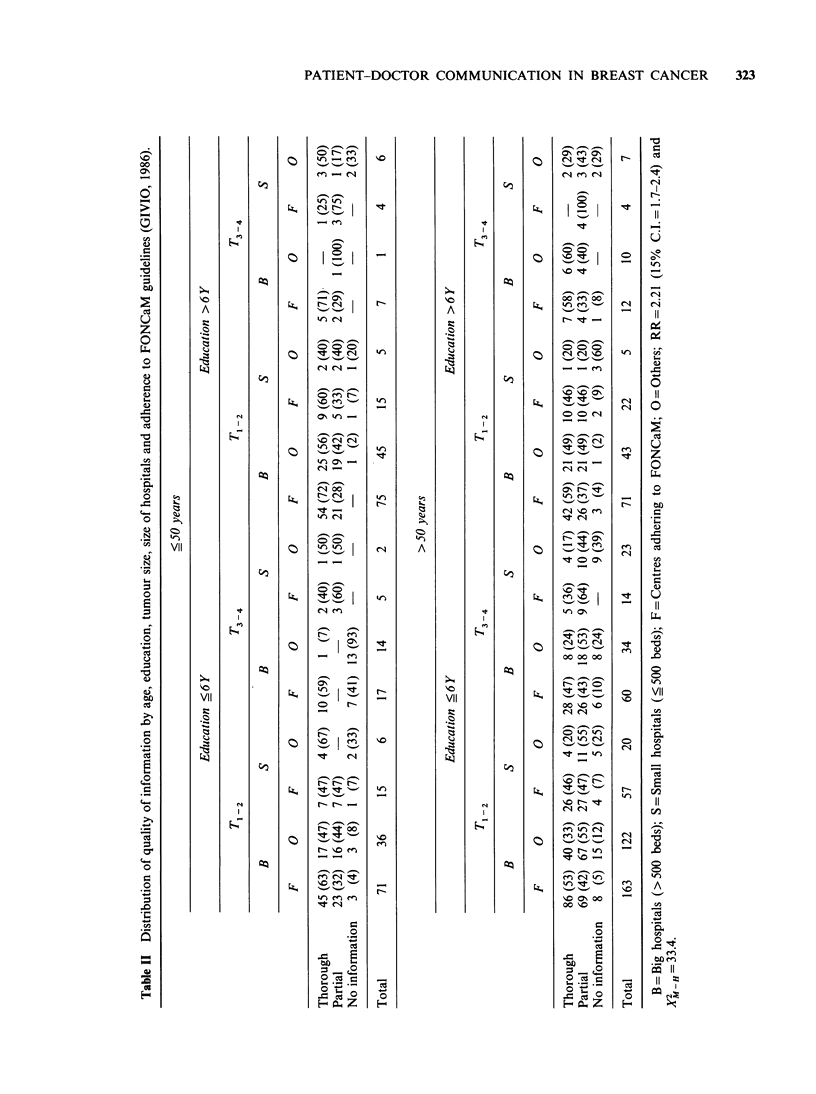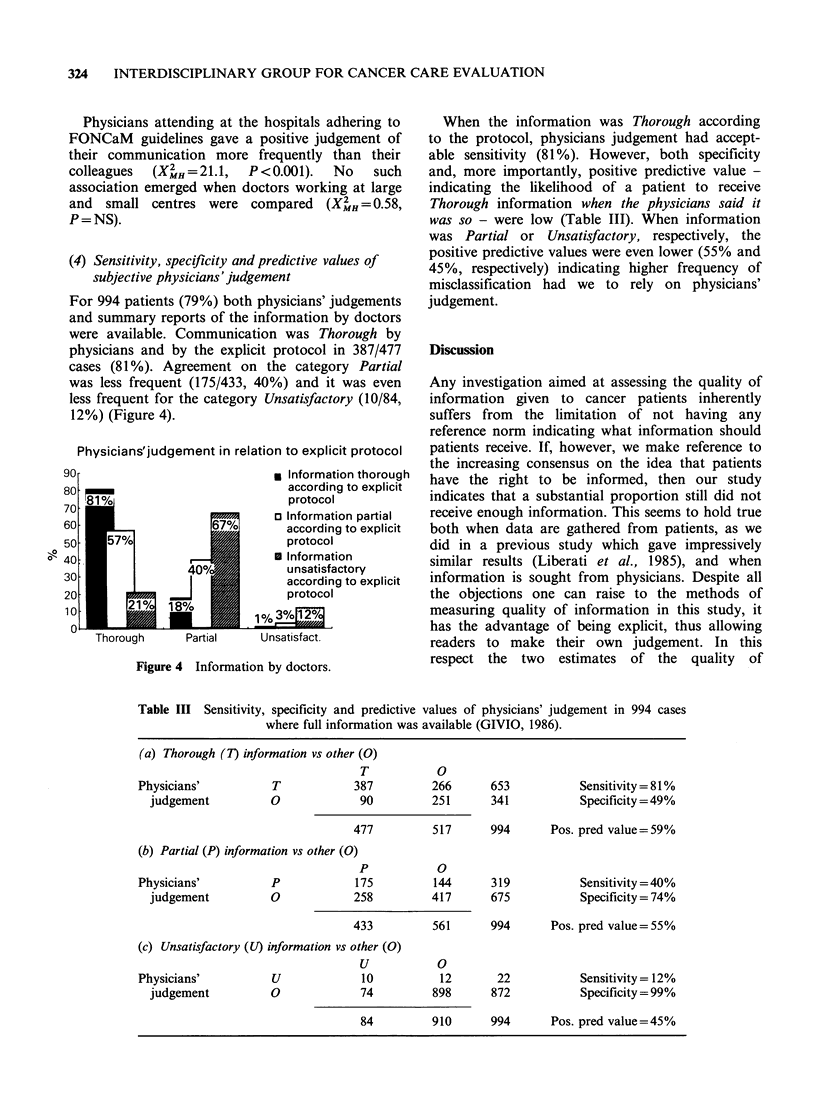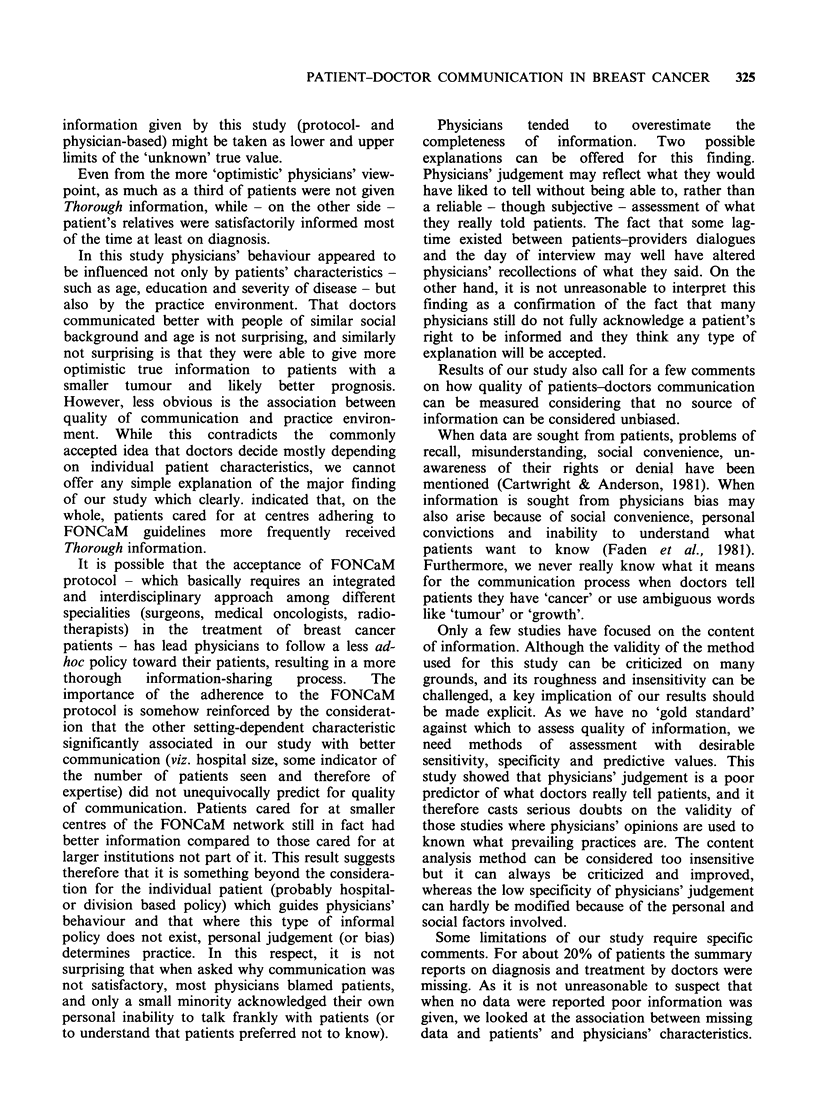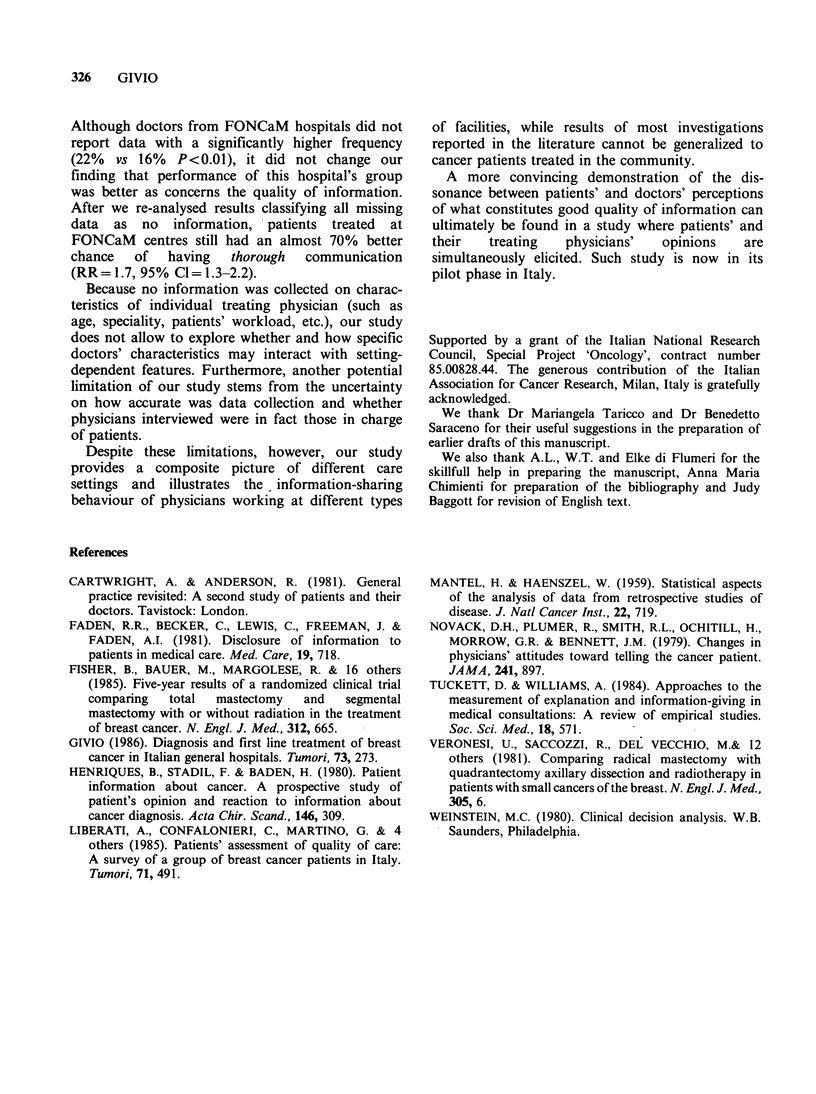# What doctors tell patients with breast cancer about diagnosis and treatment: findings from a study in general hospitals. GIVIO (Interdisciplinary Group for Cancer Care Evaluation) Italy.

**DOI:** 10.1038/bjc.1986.179

**Published:** 1986-08

**Authors:** 

## Abstract

In a study aimed at assessing whether and how patients with breast cancer are informed on their diagnosis and treatment a large group of physicians participating in a quality of care evaluation program were asked to report what they told patients about diagnosis and treatment. The completeness of such communication was then assessed using an explicit protocol designed to measure precision and lack of ambiguity of reported phrases. By this measure 39% patients received 'thorough' information on diagnosis and 11% 'detailed' information on surgery. These proportions become 48% and 14%, respectively, when only cases for whom answers were available are considered. Physicians, however, considered this communication 'thorough' for 69% of patients. Among patient-related characteristics, age, education and stage of disease were independent predictors of quality of information. Setting-dependent features more than individual provider attitudes seemed to account for at least part of the quality of information sharing behaviour as both hospital size (comparing centres larger than 500 beds and smaller ones) and degree of hospital organization (comparing centres adhering to the Italian Breast Cancer Task Force, FONCaM and those not) were - simultaneously - significant predictors of quality of communication, independently from patients' case-mix. Physicians' judgement - measured assuming the explicit protocol as standard - proved to be of acceptable sensitivity only when information was 'Thorough' by the protocol. However, its specificity and predictive values were consistently low in all three categories defined by the protocol, leading to high misclassification rates. The implications of these findings for studies aimed at assessing the quality of patients-providers communication are discussed.


Br. J. Cancer (1986), 54, 319–326

### What doctors tell patients with breast cancer about diagnosis and treatment: Findings from a study in general hospitals

GIVIO* (Interdisciplinary Group for Cancer Care Evaluation) Italy.

**Summary** In a study aimed at assessing whether and how patients with breast cancer are informed on their diagnosis and treatment a large group of physicians participating in a quality of care evaluation program were asked to report what they told patients about diagnosis and treatment. The completeness of such communication was then assessed using an explicit protocol designed to measure precision and lack of ambiguity of reported phrases.

By this measure 39% patients received 'thorough' information on diagnosis and 11% 'detailed' information on surgery. These proportions become 48% and 14%, respectively, when only cases for whom answers were available are considered. Physicians, however, considered this communication 'thorough' for 69% of patients. Among patient-related characteristics, age, education and stage of disease were independent predictors of quality of information. Setting-dependent features more than individual provider attitudes seemed to account for at least part of the quality of information sharing behaviour as both hospital size (comparing centres larger than 500 beds and smaller ones) and degree of hospital organization (comparing centres adhering to the Italian Breast Cancer Task Force, FONCaM and those not) were – simultaneously – significant predictors of quality of communication, independently from patients' case-mix. Physicians' judgement – measured assuming the explicit protocol as standard – proved to be of acceptable sensitivity only when information was 'Thorough' by the protocol. However, its specificity and predictive values were consistently low in all three categories defined by the protocol, leading to high misclassification rates. The implications of these findings for studies aimed at assessing the quality of patients–providers communication are discussed.

Although it is becoming generally accepted that cancer patients have the right to be adequately informed, many physicians tacitly assume that they fare better in ignorance (Henriques *et al.*, 1980). Most studies on the information sharing process have been made in the US where concern regarding the application of the informed consent law and its impact on general practice has prompted several investigations.

Overall those studies indicated that physicians' attitudes are changing and most of them are now willing to tell cancer patients their diagnosis and inform them of possible therapeutic alternatives (Novack *et al.*, 1979). Only a few studies have been

Correspondence: A. Liberati, Mario Negri Institute for Pharmacological Research, Via Eritrea 62, 20157 Milan, Italy.

Received 17 February 1986; and in revised form 1 April 1986.

**Members of the GIVIO coordinating centre* (Mario Negri Institute) *and writing committee:* A. Liberati, R. Fossati, R. Talamini, F. Parazzini, C. Confalonieri (Rho Hospital), S. Marsoni, W. Torri and G. Tognoni.

*Clinicians from the following hospitals who are co-authors of this paper:* Alba (*P. Gosso, D. Tagliati*); Andria (*F. Porzio*); Aosta (*F. Di Vito, F. Grasso*); Arezzo (*P.A. Nannicini, M. Rinaldini*); Assisi (*G. Di Biagio*); Avellino (*M. Belli, G. Colantuoni*); Benevento (*T. Pedicini, R. Vincenti*); Bergamo (*M. Fumagalli, G. Gritti*); Biella (*M.P. Sala Hugo*); Bollate (*E. Piatto, A. Giovaninetti*); Bologna (*A. Neri, A.M. Jannini*); Brescia (*G. Marini, A. Zaniboni*); Busto Arsizio (*C. Massazza, C. Ravetto*); Cagliari (*A. Tarquini, P. Onnis*); Cantù (*M. Casiraghi*); Carmagnola (*R. Fugaldi, U. Lucci Chiarissi*); Castelfranco Veneto (*P. Manente*); Castiglion del Lago (*M. Moretti*); Colorno (*A. Rossetti, S. Castello*); Cuggiono (*M. Potestio*); Cuneo (*D. Perroni*); Cuorgnè (*F. Peradotto*); Faenza (*A. Gambi, F. Foglietta*); Fano (*G. Nicotra, V. Saba*); Foggia (*M. Angiolillo*); Forli (*R. Ridolfi*); Formia (*G. Cardi*); Gorgonzola (*A. Silvani, R. Scapaticci*); Gorizia (*E. Benedetti*); Iseo (*R. Pedretti*); Lecco (*E. Corti*); Legnano (*A. Assi, G. Sbarbaro*); Lucca (*A. Sargenti*); Magenta (*F. Lombardi, M.E. Faccendini*); Mantova (*H. Pini, F. Smerieri*); Mestre (*O. Nascimben, L. Griggio*); Niguarda-Milano (*O. Gottardi, L. Franchi*); San Carlo-Milano (*D. Tabiadon*); Monfalcone (*A. Governa*); Padova (*D. Nitti*); Perugia (*F. Roila, R. Cristofani*); Pontedera (*M. Castiglioni, G. Di Grazia*); Pordenone (*E. Galligioni*); Potenza (*G. Elifani*); Ravenna (*A. Tienghi*); Reggio Emilia (*L. Armaroli*); Rho (*P. Viola*); Roma (*E. Cortesi*); Salò (*M. De Giuli*); San Donà di Piave (*C. D'Atri, P. Boccato*); S. Vito al Tagliamento (*R. Plaino, F. Buda*); Savigliano (*L. Galletto*); Sondalo (*L. Trimarchi*); Sondrio (*G. Gherardi*); Susa (*R. Sapuppo, G. Balocco*); Terni (*F. Di Costanzo, D. Padalino*); Tirano (*B. Trolli, A. Casagrande*); S. Anna-Torino (*A. Rolfo, G. Vaudano*); S. Giovanni-Torino (*F. Fracchia, A. Boidi Trotti*); Udine (*A. Rosa Bian*); Varese (*D. Cosentino*); Varzi (*F. Battista*); Venezia (*G. Fila*).

© The Macmillan Press Ltd., 1986

made in Europe where the lack of legal obligation leaves to the individual physician the decision whether or not to inform patient(s).

Among those favouring sharing of information between patients and physicians are those who say that patients are dissatisfied with the amount they now receive, that satisfaction with information can improve patients' compliance to medical advice, and that it is the patient who has to take the ultimate decision among the several treatment options today available (Tuckett & Williams, 1984).

On the other hand, there are also sceptical views about the feasibility and utility of free exchange of information between patients and doctors. There is, for instance, a widespread belief among doctors that patients forget, or deny, much of what they are told or that they are often unable to understand information, or that a rational exchange of information could negatively affect patients' trust in physicians, thus undermining therapeutic effectiveness (Tuckett & Williams, 1984).

In Italy no large investigations have been made on whether and how cancer patients receive adequate information. A first step in this direction was taken in 1983 with a mail survey to a large group of breast cancer patients to assess whether they had been informed about diagnosis and treatment and whether the lack of this information caused dissatisfaction (Liberati *et al.*, 1985). The study showed that only about a third (37%) of the patients received thorough information but that only 18% complained for this lack of communication. We have now addressed the same question from a different perspective and interviewed each patient's treating physician in the framework of a study on quality of breast cancer care (GIVIO, 1986) asking them to report what they told patients about diagnosis and treatment and how satisfactory they consider such information.

Analysis of what doctors reportedly said to their patients indicated that a substantial proportion of cases did not receive satisfactory information. Moreover, we found substantial evidence that physicians tend to overestimate the completeness of such information. The paper discusses the implications of these findings with respect to how the quality of patient–physician communication should be evaluated.

### Methods

Data presented here are part of a larger prospective study on quality of breast cancer care in Italian general hospitals; detailed methodology and data collection procedures have been reported elsewhere (GIVIO, 1986). Information was collected in each of the 62 participating hospitals by a medically qualified investigator using standard forms. Besides a detailed clinical and demographic description, the study protocol required in the charts a summary of what doctors told patients and their relatives about diagnosis and surgical treatment, retrieved by personal interview with each patient's treating physician. In addition, doctors were asked for their own personal judgment on the quality of this communication, rating it as: (a) *Satisfactory*, (b) *Partial*, (c) *Unsatisfactory*. According to the study protocol, interviews were conducted during the patient's first admission with a lag-time between doctor–patient dialogue and interview ranging from 1 to 30 days.

Summaries of patient–doctor communication relative to diagnosis and treatment were centrally analyzed by two of us (R.F. and R.T.) rating phrases reported in each patient's form according to a predefined explicit protocal already tested in a study where quality of information was explored from patients.

Quality was classified using a 3-level categorical scale (*Thorough, Partial, No information*) based on the following rules. When physicians' phrases included words like: 'breast cancer, tumours, cancer neoplasm malignant nodule' the information was classified *Thorough*. When words like 'benign nodule, lesion of borderline nature, benign tumour' were reported information was classified *Vague* or *Partial*. Finally, when 'nothing was reported in the questionnaire, or the content did not fall in either of the previous categories', information was classifed *No information*.

The quality of information about surgery was also assessed using a pre-defined protocol. When physicians' reports clearly indicated that patients have been told that a mastectomy plus axillary dissection was required, information was classified *Detailed* and when physicians' reports showed that patients were told that mastectomy was required without further information it was classified *Acceptable*. Finally, when only vague reference was made to the fact some surgery would be required without specifying the type and extent in physicians' statements, information was classified *Unsatisfactory*.

Quantitative estimates of the effects of patient- and hospital-related characteristics on the quality of information were obtained using odds ratios (as estimates of the relative risk, RR's) and their 95% confidence intervals (CI). The test of statistical significance for contingency tables was based on the usual chi-square value comparing observed and expected numbers of events. The potential reciprocal confouding effects of patients' age, education, disease stage, hospitals' size and degree of specialization were controlled for using stratification and the Mantel–Haenszel procedure (Mantel & Haenszel, 1959). All relative risks estimates presented in the text are those simultaneously adjusted for all the above covariates.

When quality of information was analyzed with reference to characteristics of hospitals where doctors practised, centres were grouped according to the following features; (a) *Size* (<500 *vs.* >500 beds); (b) *Presence or absence of oncologic departments/wards*; (c) *reported adherence to the Italian Breast Cancer Task Force* (*FONCaM*) *guidelines*.

Estimates of sensitivity, specificity, and positive predictive values reported in the text were computed according to the classic method reported by Weinstein *et al.* (1980). Calculations have been done assuming physicians' judgment as diagnostic test and quality assessment by the explicit protocol as standard.

### Results

### (*1*) *Characteristics of patient population*

This study refers to the care offered to 1262 newly diagnosed breast cancer patients whose general characteristics are reported in Table I. Most patients (893, 71%) were older than 50 years, 67% ($n = 843$) had less than 6 years of education and 66% ($n = 833$) were married. At diagnosis, 949 (75%) patients had a primary lesion smaller than 5 cm (T1 and T2 according to the TNM system) and the following distribution in terms of clinical stage: *Stage I*; 224 patients (18%), *Stage II*; 582 (46%), *Stage III*; 258 (20%), *Stage IV*; 27 (2%); for 171 cases (14%) this information was not available. Patients accrual rate differed among the 62 participating hospitals with 12 (19%) hospitals contributing 40 or more cases, 4 (6%) centres between 30 and 39 patients, 10 (16%) between 20 and 29, 25 (40%) between 10 and 19, and 11 hospitals (18%) with less than 10 patients.

### (*2*) *Explicit assessment of completeness of communication*

Figure 1 shows the frequency distribution of the information on diagnosis and surgical treatment (Figure 2) according to the explicit protocol. *Thorough* information on diagnosis was given to 488 (39%) women, *vague* to 443 (35%) while *no information* to 84 (7%). If missing data (247, 20%) are excluded from the denominator the above proportions become 48%, 43% and 8%, respectively. *Detailed* information on surgery was given to 135 patients (11%), *acceptable* to 693 (55%) and *vague* to 167 (13%). Without missing data (267, 21%) proportions become respectively 14%, 69% and 17%. If we consider patients who received *Thorough* information on diagnosis and *Detailed* or *Acceptable* information on surgery, proportions are 31% (389/1262) if all patients are considered, and 42% (389/924) when missing data are excluded. Data on information given to patients' relatives was not reported by almost half of participating physicians. Among 52% responders, however, information was rated *Thorough* in 91% on diagnosis, and *Detailed* in 23% or *Acceptable* in 62% relative to surgical treatment.

Among patients' characteristics, age education

**Table I** Characteristics of the 1262 patients with breast cancer enrolled in the quality of care study (GIVIO, 1986).

| *Age* (*yrs*) | *No.* (%) | *Education* (*yrs*) | *No.* (%) | *Marital status* | *No.* (%) |
|---|---|---|---|---|---|
| >30 | 5 (-) | | | | |
| 31–50 | 364 (29) | <6 | 843 (67) | Ever married | 833 (66) |
| 51–60 | 311 (25) | 6–8 | 193 (15) | Never married | 160 (13) |
| 61–70 | 299 (24) | ≥9 | 127 (10) | Widowed | 266 (21) |
| >70 | 283 (22) | N.E. | 99 (8) | Divorced | |
| | | | | N.E. | 3 (—) |

| *Size of primary tumour at diagnosis* | *No.* (%) | *Clinical stage at diagnosis* | *No.* (%) | *Performance status* (*ECOG*) *P.S.* | *No.* (%) |
|---|---|---|---|---|---|
| T1 | 335 (27) | I | 224 (18) | 0 | 1073 (85) |
| T2 | 614 (49) | II | 582 (46) | 1 | 153 (12) |
| T3 | 111 (9) | III | 258 (20) | 2 | 20 (2) |
| T4 | 182 (14) | IV | 27 (2) | 3 | 11 (1) |
| N.E. | 20 (1) | N.E. | 171 (14) | 4 | 5 (—) |

**Figure 1** Quality distribution of information on diagnosis.

**Figure 2** Quality distribution of information on surgery.

and size of the tumour were independent significant predictors of quality of information of diagnosis. Younger (<50 years) and more educated women (>6 years of education) were in fact about 50% more likely (RR = 1.5, 95% Cl = 1.1–2.0 and RR = 1.4, 95% Cl = 1.1–1.9, respectively) to receive *thorough* information than older and less educated patients. Women whose tumour was smaller than 5 cm at diagnosis had a similarly greater chance (RR = 1.5, 95% Cl = 1.0–2.0) of receiving better information. When the possible relationship between quality of information and setting-dependent characteristics was explored, interesting associations emerged. Larger and more organized centres (i.e. those adhering to FONCaM guidelines) seemed to predict for women receiving more complete information, independently of the patients' case-mix. Patients treated at larger centres were in fact almost 60% more likely (RR = 1.6, 95% Cl = 1.2–2.2) to have better information. When patients treated at centres adhering to FONCaM were compared to those treated elsewhere, a stronger association emerged. Women cared for at more organized centres had in fact an almost doubled chance of receiving thorough information (RR = 2.2, 95% Cl = 1.7–2.9) (Table II).

Finally, to test the hypothesis that patients who share better information with doctors are – if eligible – more likely to receive limited surgery, we compared those who had quadrantectomy with those who had more radical surgery among groups identified by Veronesi's (Veronesi *et al.*, 1981) and Fisher's (Fisher *et al.*, 1985) eligibility criteria for conservative surgery. After allowing for the potential confounding role of patients' age, education and disease stage – in addition to hospital size and degree of organization – results showed no consistent pattern for any better quality of information.

### (3) *How physicians perceived the completeness of information*

When asked to evaluate their communication with patients, physicians rated it as *Thorough* in 788 cases (62%), *Partial* in 374 (30%), and *Unsatisfactory* for 32 patients (3%); data were missing for 68 (5%) patients (Figure 3). Doctors were also asked to say what prevented them from a satisfactory disclosure of the truth. While 58% of physicians did not answer this question, the vast majority (80%) of responders referred to patients' psychological problems and 11% to their limited education. Only 9% of doctors acknowledged their own personal inability to communicate frankly with patients.

When the perceived quality of information was analysed for association with patients' and doctors' characteristics, the picture was similar to the one from the explicit assessment. Doctors in fact felt they had been able to give better information to more educated patients ($X^2_{MH} = 28.4$, $P < 0.001$) and – independently from that – to those with earlier disease ($X^2_{MH} = 35.3$, $P < 0.001$); there was no difference in the perceived quality of information when patients were grouped according to the type of surgery (radical *vs.* conservative).

**Figure 3** Physicians' perception of quality of information.

**Table II** Distribution of quality of information by age, education, tumour size, size of hospitals and adherence to FONCaM guidelines (GIVIO, 1986).

| | ≦50 years | | | | | | | | | | | | | | | |
|---|---|---|---|---|---|---|---|---|---|---|---|---|---|---|---|---|
| | Education ≦6Y | | | | | | | | Education >6Y | | | | | | | |
| | $T_{1-2}$ | | | | $T_{3-4}$ | | | | $T_{1-2}$ | | | | $T_{3-4}$ | | | |
| | B | | S | | B | | S | | B | | S | | B | | S | |
| | F | O | F | O | F | O | F | O | F | O | F | O | F | O | F | O |
| Thorough | 45 (63) | 17 (47) | 7 (47) | 4 (67) | 10 (59) | 1 (7) | 2 (40) | 1 (50) | 54 (72) | 25 (56) | 9 (60) | 2 (40) | 5 (71) | — | 1 (25) | 3 (50) |
| Partial | 23 (32) | 16 (44) | 7 (47) | — | — | — | 3 (60) | 1 (50) | 21 (28) | 19 (42) | 5 (33) | 2 (40) | 2 (29) | 1 (100) | 3 (75) | 1 (17) |
| No information | 3 (4) | 3 (8) | 1 (7) | 2 (33) | 7 (41) | 13 (93) | — | — | — | 1 (2) | 1 (7) | 1 (20) | — | — | — | 2 (33) |
| Total | 71 | 36 | 15 | 6 | 17 | 14 | 5 | 2 | 75 | 45 | 15 | 5 | 7 | 1 | 4 | 6 |

| | >50 years | | | | | | | | | | | | | | | |
|---|---|---|---|---|---|---|---|---|---|---|---|---|---|---|---|---|
| | Education ≦6Y | | | | | | | | Education >6Y | | | | | | | |
| | $T_{1-2}$ | | | | $T_{3-4}$ | | | | $T_{1-2}$ | | | | $T_{3-4}$ | | | |
| | B | | S | | B | | S | | B | | S | | B | | S | |
| | F | O | F | O | F | O | F | O | F | O | F | O | F | O | F | O |
| Thorough | 86 (53) | 40 (33) | 26 (46) | 4 (20) | 28 (47) | 8 (24) | 5 (36) | 4 (17) | 42 (59) | 21 (49) | 10 (46) | 1 (20) | 7 (58) | 6 (60) | — | 2 (29) |
| Partial | 69 (42) | 67 (55) | 27 (47) | 11 (55) | 26 (43) | 18 (53) | 9 (64) | 10 (44) | 26 (37) | 21 (49) | 10 (46) | 1 (20) | 4 (33) | 4 (40) | 4 (100) | 3 (43) |
| No information | 8 (5) | 15 (12) | 4 (7) | 5 (25) | 6 (10) | 8 (24) | — | 9 (39) | 3 (4) | 1 (2) | 2 (9) | 3 (60) | 1 (8) | — | — | 2 (29) |
| Total | 163 | 122 | 57 | 20 | 60 | 34 | 14 | 23 | 71 | 43 | 22 | 5 | 12 | 10 | 4 | 7 |

B = Big hospitals (>500 beds); S = Small hospitals (≦500 beds); F = Centres adhering to FONCaM; O = Others; RR = 2.21 (15% C.I. = 1.7–2.4) and $X^2_{M-H} = 33.4$.

Physicians attending at the hospitals adhering to FONCaM guidelines gave a positive judgement of their communication more frequently than their colleagues ($X^2_{MH}=21.1$, $P<0.001$). No such association emerged when doctors working at large and small centres were compared ($X^2_{MH}=0.58$, $P=$NS).

*(4) Sensitivity, specificity and predictive values of subjective physicians' judgement*

For 994 patients (79%) both physicians' judgements and summary reports of the information by doctors were available. Communication was *Thorough* by physicians and by the explicit protocol in 387/477 cases (81%). Agreement on the category *Partial* was less frequent (175/433, 40%) and it was even less frequent for the category *Unsatisfactory* (10/84, 12%) (Figure 4).

**Figure 4** Information by doctors.

When the information was *Thorough* according to the protocol, physicians judgement had acceptable sensitivity (81%). However, both specificity and, more importantly, positive predictive value – indicating the likelihood of a patient to receive *Thorough* information *when the physicians said it was so* – were low (Table III). When information was *Partial* or *Unsatisfactory,* respectively, the positive predictive values were even lower (55% and 45%, respectively) indicating higher frequency of misclassification had we to rely on physicians' judgement.

### Discussion

Any investigation aimed at assessing the quality of information given to cancer patients inherently suffers from the limitation of not having any reference norm indicating what information should patients receive. If, however, we make reference to the increasing consensus on the idea that patients have the right to be informed, then our study indicates that a substantial proportion still did not receive enough information. This seems to hold true both when data are gathered from patients, as we did in a previous study which gave impressively similar results (Liberati *et al.,* 1985), and when information is sought from physicians. Despite all the objections one can raise to the methods of measuring quality of information in this study, it has the advantage of being explicit, thus allowing readers to make their own judgement. In this respect the two estimates of the quality of

**Table III** Sensitivity, specificity and predictive values of physicians' judgement in 994 cases where full information was available (GIVIO, 1986).

| | | | | | |
|---|---|---|---|---|---|
| *(a) Thorough (T) information vs other (O)* | | | | | |
| | | *T* | *O* | | |
| Physicians' | *T* | 387 | 266 | 653 | Sensitivity = 81% |
| judgement | *O* | 90 | 251 | 341 | Specificity = 49% |
| | | 477 | 517 | 994 | Pos. pred value = 59% |
| *(b) Partial (P) information vs other (O)* | | | | | |
| | | *P* | *O* | | |
| Physicians' | *P* | 175 | 144 | 319 | Sensitivity = 40% |
| judgement | *O* | 258 | 417 | 675 | Specificity = 74% |
| | | 433 | 561 | 994 | Pos. pred value = 55% |
| *(c) Unsatisfactory (U) information vs other (O)* | | | | | |
| | | *U* | *O* | | |
| Physicians' | *U* | 10 | 12 | 22 | Sensitivity = 12% |
| judgement | *O* | 74 | 898 | 872 | Specificity = 99% |
| | | 84 | 910 | 994 | Pos. pred value = 45% |

information given by this study (protocol- and physician-based) might be taken as lower and upper limits of the 'unknown' true value.

Even from the more 'optimistic' physicians' viewpoint, as much as a third of patients were not given *Thorough* information, while – on the other side – patient's relatives were satisfactorily informed most of the time at least on diagnosis.

In this study physicians' behaviour appeared to be influenced not only by patients' characteristics – such as age, education and severity of disease – but also by the practice environment. That doctors communicated better with people of similar social background and age is not surprising, and similarly not surprising is that they were able to give more optimistic true information to patients with a smaller tumour and likely better prognosis. However, less obvious is the association between quality of communication and practice environment. While this contradicts the commonly accepted idea that doctors decide mostly depending on individual patient characteristics, we cannot offer any simple explanation of the major finding of our study which clearly. indicated that, on the whole, patients cared for at centres adhering to FONCaM guidelines more frequently received *Thorough* information.

It is possible that the acceptance of FONCaM protocol – which basically requires an integrated and interdisciplinary approach among different specialities (surgeons, medical oncologists, radiotherapists) in the treatment of breast cancer patients – has lead physicians to follow a less *ad-hoc* policy toward their patients, resulting in a more thorough information-sharing process. The importance of the adherence to the FONCaM protocol is somehow reinforced by the consideration that the other setting-dependent characteristic significantly associated in our study with better communication (*viz.* hospital size, some indicator of the number of patients seen and therefore of expertise) did not unequivocally predict for quality of communication. Patients cared for at smaller centres of the FONCaM network still in fact had better information compared to those cared for at larger institutions not part of it. This result suggests therefore that it is something beyond the consideration for the individual patient (probably hospital- or division based policy) which guides physicians' behaviour and that where this type of informal policy does not exist, personal judgement (or bias) determines practice. In this respect, it is not surprising that when asked why communication was not satisfactory, most physicians blamed patients, and only a small minority acknowledged their own personal inability to talk frankly with patients (or to understand that patients preferred not to know).

Physicians tended to overestimate the completeness of information. Two possible explanations can be offered for this finding. Physicians' judgement may reflect what they would have liked to tell without being able to, rather than a reliable – though subjective – assessment of what they really told patients. The fact that some lag-time existed between patients–providers dialogues and the day of interview may well have altered physicians' recollections of what they said. On the other hand, it is not unreasonable to interpret this finding as a confirmation of the fact that many physicians still do not fully acknowledge a patient's right to be informed and they think any type of explanation will be accepted.

Results of our study also call for a few comments on how quality of patients–doctors communication can be measured considering that no source of information can be considered unbiased.

When data are sought from patients, problems of recall, misunderstanding, social convenience, unawareness of their rights or denial have been mentioned (Cartwright & Anderson, 1981). When information is sought from physicians bias may also arise because of social convenience, personal convictions and inability to understand what patients want to know (Faden *et al.*, 1981). Furthermore, we never really know what it means for the communication process when doctors tell patients they have 'cancer' or use ambiguous words like 'tumour' or 'growth'.

Only a few studies have focused on the content of information. Although the validity of the method used for this study can be criticized on many grounds, and its roughness and insensitivity can be challenged, a key implication of our results should be made explicit. As we have no 'gold standard' against which to assess quality of information, we need methods of assessment with desirable sensitivity, specificity and predictive values. This study showed that physicians' judgement is a poor predictor of what doctors really tell patients, and it therefore casts serious doubts on the validity of those studies where physicians' opinions are used to known what prevailing practices are. The content analysis method can be considered too insensitive but it can always be criticized and improved, whereas the low specificity of physicians' judgement can hardly be modified because of the personal and social factors involved.

Some limitations of our study require specific comments. For about 20% of patients the summary reports on diagnosis and treatment by doctors were missing. As it is not unreasonable to suspect that when no data were reported poor information was given, we looked at the association between missing data and patients' and physicians' characteristics.

Although doctors from FONCaM hospitals did not report data with a significantly higher frequency (22% *vs* 16% $P<0.01$), it did not change our finding that performance of this hospital's group was better as concerns the quality of information. After we re-analysed results classifying all missing data as no information, patients treated at FONCaM centres still had an almost 70% better chance of having *thorough* communication (RR = 1.7, 95% Cl = 1.3–2.2).

Because no information was collected on characteristics of individual treating physician (such as age, speciality, patients' workload, etc.), our study does not allow to explore whether and how specific doctors' characteristics may interact with setting-dependent features. Furthermore, another potential limitation of our study stems from the uncertainty on how accurate was data collection and whether physicians interviewed were in fact those in charge of patients.

Despite these limitations, however, our study provides a composite picture of different care settings and illustrates the information-sharing behaviour of physicians working at different types of facilities, while results of most investigations reported in the literature cannot be generalized to cancer patients treated in the community.

A more convincing demonstration of the dissonance between patients' and doctors' perceptions of what constitutes good quality of information can ultimately be found in a study where patients' and their treating physicians' opinions are simultaneously elicited. Such study is now in its pilot phase in Italy.

Supported by a grant of the Italian National Research Council, Special Project 'Oncology', contract number 85.00828.44. The generous contribution of the Italian Association for Cancer Research, Milan, Italy is gratefully acknowledged.

We thank Dr Mariangela Taricco and Dr Benedetto Saraceno for their useful suggestions in the preparation of earlier drafts of this manuscript.

We also thank A.L., W.T. and Elke di Flumeri for the skillfull help in preparing the manuscript, Anna Maria Chimienti for preparation of the bibliography and Judy Baggott for revision of English text.

### References

CARTWRIGHT, A. & ANDERSON, R. (1981). General practice revisited: A second study of patients and their doctors. Tavistock: London.

FADEN, R.R., BECKER, C., LEWIS, C., FREEMAN, J. & FADEN, A.I. (1981). Disclosure of information to patients in medical care. *Med. Care,* **19,** 718.

FISHER, B., BAUER, M., MARGOLESE, R. & 16 others (1985). Five-year results of a randomized clinical trial comparing total mastectomy and segmental mastectomy with or without radiation in the treatment of breast cancer. *N. Engl. J. Med.,* **312,** 665.

GIVIO (1986). Diagnosis and first line treatment of breast cancer in Italian general hospitals. *Tumori,* **73,** 273.

HENRIQUES, B., STADIL, F. & BADEN, H. (1980). Patient information about cancer. A prospective study of patient's opinion and reaction to information about cancer diagnosis. *Acta Chir. Scand.,* **146,** 309.

LIBERATI, A., CONFALONIERI, C., MARTINO, G. & 4 others (1985). Patients' assessment of quality of care: A survey of a group of breast cancer patients in Italy. *Tumori,* **71,** 491.

MANTEL, H. & HAENSZEL, W. (1959). Statistical aspects of the analysis of data from retrospective studies of disease. *J. Natl Cancer Inst.,* **22,** 719.

NOVACK, D.H., PLUMER, R., SMITH, R.L., OCHITILL, H., MORROW, G.R. & BENNETT, J.M. (1979). Changes in physicians' attitudes toward telling the cancer patient. *JAMA,* **241,** 897.

TUCKETT, D. & WILLIAMS, A. (1984). Approaches to the measurement of explanation and information-giving in medical consultations: A review of empirical studies. *Soc. Sci. Med.,* **18,** 571.

VERONESI, U., SACCOZZI, R., DEL VECCHIO, M.& 12 others (1981). Comparing radical mastectomy with quadrantectomy axillary dissection and radiotherapy in patients with small cancers of the breast. *N. Engl. J. Med.,* **305,** 6.

WEINSTEIN, M.C. (1980). Clinical decision analysis. W.B. Saunders, Philadelphia.